# Creation of a telehealth addiction consultation service at a rural hospital: a case study

**DOI:** 10.1186/s13722-025-00596-5

**Published:** 2025-09-26

**Authors:** Rachel Katz, Tiarra Fisher, Talia Singer-Clark, William Soares III, Jane Carpenter, Nadia Schuessler, Henry Stadler, Andrea Sahovey, Ann Scheck McAlearney, Jeffrey H. Samet, Avik Chatterjee

**Affiliations:** 1 Independent Researcher, Greenfield, MA USA; 2 The Opioid Task Force of Franklin County and the North Quabbin Region, Franklin, USA; 3 Boston Medical Center, Section of General Internal Medicine, Department of Medicine, Boston University Chobanian & Avedisian School of Medicine, Boston, MA USA; 4 Department of Emergency Medicine, UMASS Chan Medical School, Baystate, USA; 5 Institute for Behavioral Medicine Research, The Ohio State University, Columbus, OH USA

**Keywords:** Opioid, Overdose, Rural, Medications for opioid use disorder, Addiction consult service

## Abstract

**Background:**

Rural communities face significant barriers to accessing substance use disorder (SUD) treatment, resulting in gaps in care and increased rates of opioid-related overdose deaths. Hospital-based Addiction Consult Services (ACS) improve outcomes for patients with SUD, but rural hospitals often lack these services.

**Case presentation:**

The Community Addiction Consult (CAC) service was established at a rural hospital in western Massachusetts to address this gap. CAC was designed by a community coalition comprised of a diverse cross-section of the community in which the hospital is based, using opioid-overdose data from the region to inform their decisions. Using a telehealth model, the CAC provided evidence-based treatments to support hospital staff treating patients with opioid use disorder (OUD) or requiring addiction-related care. From April 2023 through December 2023, the CAC provided 36 consults, facilitating increased access to medications for opioid use disorder (MOUD), and enhancing provider confidence in treating people who use drugs (PWUD) and initiating MOUD. An average of 22 patients received MOUD as inpatients monthly, and 11 emergency department patients received MOUD monthly. The CAC team also implemented training sessions, and an anti-stigma campaign to familiarize hospital staff with harm reduction principles and person-centered care strategies to foster a more supportive treatment environment for PWUD.

**Conclusions:**

The Community Addiction Consult service demonstrates the feasibility and efficacy of a telehealth Addiction Consult Service model. Paired with staff trainings, such a model can bridge the gaps in rural addiction care. By leveraging local expertise and data-driven approaches, this model offers a scalable, equitable solution to improving access to substance use disorder treatment in rural settings.

## Background

Opioid-related overdose deaths continue to be a major public health problem in the United States and disproportionately impact rural communities [1]. The HEALing (Helping to End Addiction Long-term) Communities Study (HCS) is a multi-site study in 67 communities in four states (Kentucky, Massachusetts, New York and Ohio) that used the Communities that HEAL (CTH) intervention with the goal of reducing opioid overdose deaths. The Franklin County/North Quabbin (FCNQ) cluster is an HCS Massachusetts community cluster comprised of the four towns of Greenfield, Montague, Athol, and Orange. Franklin County is the only federally designated rural community in mainland Massachusetts. Though overdose death rates in Massachusetts have decreased more recently [2], the rate of fatal overdoses in Franklin County, MA, nearly doubled from 2019 to 2021, when the HCS study began, with the onset of the COVID-19 pandemic and an increasingly dangerous drug supply [3]. As a regional, rural facility, the hospital in Franklin County serves a diverse population of patients from at least 30 municipalities across the western central region of Massachusetts. The medical center is a critical point of addiction care in the region due to the lack of community-based substance use treatment options. According to the Massachusetts Bureau of Substance Addiction Services, the average resident of Franklin County has to travel 20 miles to access addiction treatment services [4]. The only acute treatment center or clinical stabilization available in the county is for women who have been legally mandated to attend treatment. Accessing treatment outside the community is limited by transportation and financial barriers. Despite Franklin County having clinicians with addiction expertise, people who use drugs (PWUD) are not regularly receiving the care that they need, including access to medications for opioid use disorder (MOUD) and harm reduction services.

Few treatment options for substance use disorders (SUDs) are accessible to rural communities and existing barriers such as transportation exacerbate the disparities in actual treatment options between rural and urban settings [5, 6]. Hospital-based Addiction Consult Services (ACS) have been demonstrated to improve clinical outcomes [7], though research has primarily focused on urban settings. Research suggests rural hospitals are less likely to use evidence-based treatment models such as ACS and MOUD [5]. MOUD are associated with lower mortality among people at high risk for overdose death [8]. While addiction consult service models can vary in practice, they share the same transformative goals to “improve preparedness among health care providers in managing SUDs, reducing stigma associated with the condition, and ultimately improving clinical practice.” [9] ACS programs have demonstrated success across the U.S. at increasing patient engagement with addiction treatment through evidenced-based practice, among other benefits [9]. We present a case study of a novel Community Addiction Consult (CAC) service at a hospital in rural western Massachusetts created as part of the HEALing Communities Study.

## Case presentation

### Community process

The CAC service implemented an addiction consultation service to increase the quality of in-hospital care for people who use drugs, increase engagement in addiction treatment services for PWUD, prevent opioid-related overdoses, and increase the knowledge and confidence of hospital staff to treat patients with substance use disorders through evidence-based training and technical assistance. The intervention model was designed by the FCNQ community coalition members following a review of relevant opioid-related overdose data. In keeping with HCS’s commitment to equity [10], coalition members were a diverse cross-section of the overall FCNQ community, including people with lived experience using drugs, members of law enforcement, and those who work in healthcare and other various supportive sectors. After reviewing local opioid-related overdose data, community members identified gaps in availability and accessibility to treatment services for persons who use drugs.

### Goals of CAC

Among other strategies selected and implemented by the coalition (e.g., a program to support transportation to addiction treatment and naloxone distribution to corrections-involved individuals), coalition members created the CAC to decrease opioid-related overdose deaths by providing low-barrier access to MOUD for individuals seeking emergency department or inpatient care in addition to improving the quality of care and outcomes for anyone using drugs or alcohol.

### Implementation process and logistics of the team

The consult team was composed of five clinicians with a combined total of 50 years of addiction clinical experience. The clinicians included four physicians, all board-certified in addiction medicine, and a nurse practitioner, all who have outpatient or other roles in the community, such as outpatient primary care, addiction medicine, or emergency medicine. Given that addiction consults sought in this rural community hospital are less frequent, the classic inpatient ACS multidisciplinary team would not be justified. Thus an alternative model was adopted in which the CAC clinical team members were on call for weekly rotations. They were hired as “per diem” employees by the hospital, paid per shift, and could access the hospital’s electronic medical record to view patient records at the time of the consult. They are available 24/7 and use a HIPAA-compliant and secure text message platform. The services provided by the CAC include telephone consultation for hospital staff focused on the diagnosis and inpatient management of SUD, the initiation and guidance for ongoing management of medications for these disorders, and discharge planning. CAC team members do not provide direct clinical services to patients and do not interact with patients directly. Independent from HCS funding, a community non-profit provided support to clinicians in seeking out care transitions, such as acute treatment services (“detox”) or inpatient residential treatment beds.

### Broader education campaign

The CAC team also launched an anti-stigma campaign through communication materials and on-site training. Hospital staff were trained on harm reduction principles, person-centered language, and supporting PWUD. Department heads and hospital leadership were required to attend an in-person training facilitated by a local harm reduction organization. Additionally, all hospital staff were encouraged to attend virtual trainings hosted by Boston Medical Center’s Grayken Center for Addiction Training and Technical Assistance and were asked to sign the “Words Matter” pledge, a commitment to treat people with SUD with dignity and respect by using non-stigmatizing language. Department heads and hospital leadership were charged with disseminating teachings from the trainings to their staff.

### Time period

The study intervention period was from January 1st, 2023, to December 31st, 2023. Following the planning and design phases, the CAC launched in April 2023. The CAC team provided consults to hospital staff treating patients with a history of drug use or overdose, a diagnosis of opioid use disorder (OUD), or otherwise needing specialized addiction treatment in a hospital setting.

### Outcomes

**Fig. 1:**
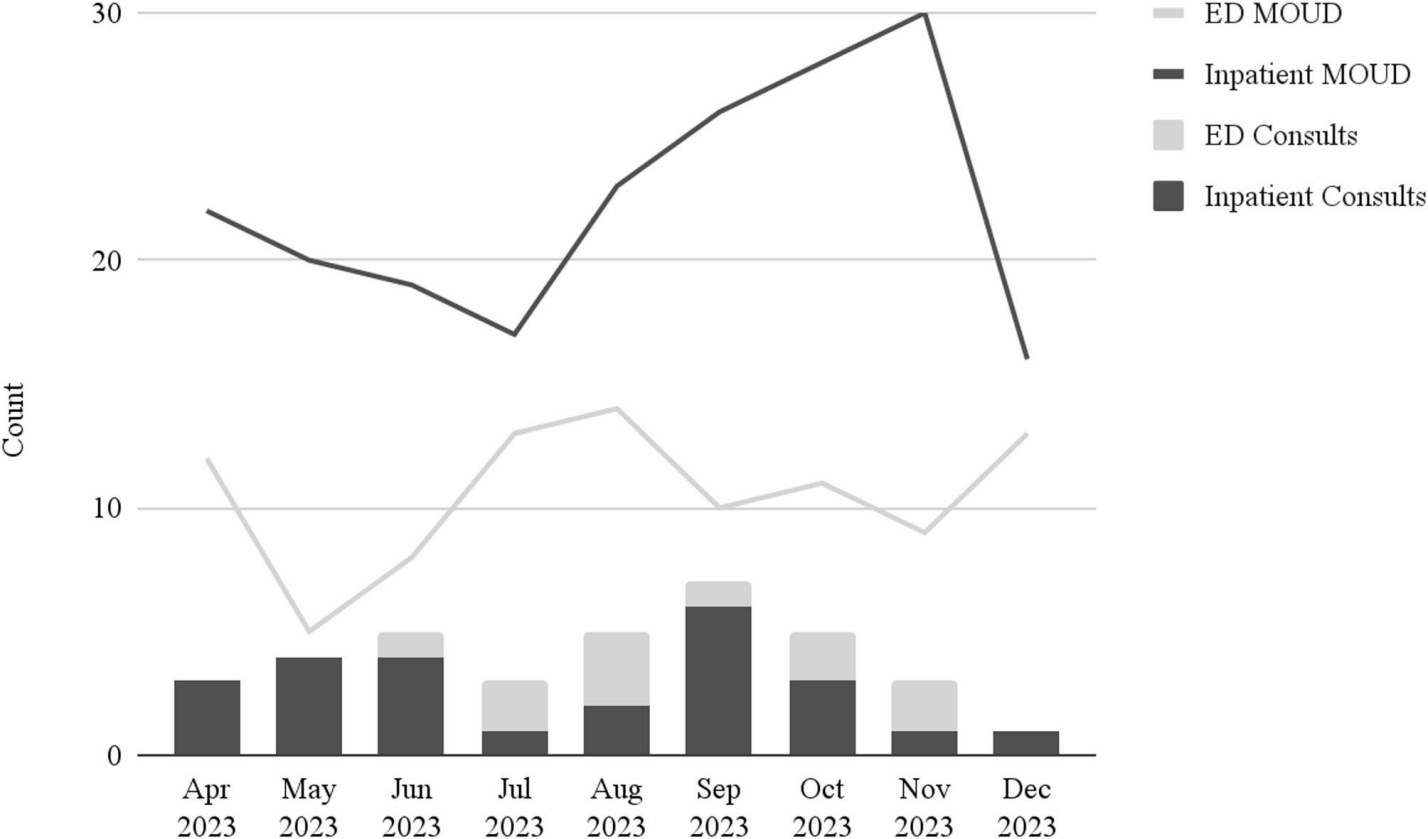
Consults provided and patients receiving medications for opioid use disorder (MOUD) during the intervention period in hospital inpatient units and the emergency department

Community clinicians used a secure form to track information about consults provided, including the consulting department (inpatient vs. emergency department), the presence or absence of a recommendation to prescribe MOUD or other treatments, and general notes about what was discussed during the consult. From the launch of the CAC program in April 2023 through the end of the study on December 31, 2023, five clinicians provided 36 consults. Twenty-five consults were provided for inpatient units and 11 for the emergency department (Fig. 1). Patients discussed during consults included complex cases of pain management for patients already on MOUD, initiation of methadone in the emergency department per the 72-hour methadone regulation [11], and withdrawal management (Table 1). The number of patients receiving MOUD as inpatients or in the emergency department was also tracked. During the intervention period, on average, 22.3 patients per month received MOUD as inpatients and 10.6 patients per month received MOUD in the emergency department. In contrast, prior to the intervention, from January 1^st,^ 2019 to March 31st, 2023, on average of seven inpatients per month and two ED patients per month received MOUD. Due to hospital staff capacity, the MOUD data were not disaggregated by the presence or absence of an official consult, and patient outcomes were not systematically tracked.

**Table 1:** Sample of clinical topics covered during consults and descriptions of example cases

Clinical Topics	Example Case Description
Pain management	Complex patient on chronic opiates including methadone for history of back pain, admitted for emergency surgery and requiring additional pain management. Addiction Consult Service (ACS) clinician consulted for inpatient pain management and tapering back to baseline doses.
Initiation of methadone in the Emergency Department	Patient presented to the emergency department requesting detox and bed placement. Based on patient’s history and current use, ACS provider recommended to emergency department clinician that patient start methadone, both for comfort dosing to avoid withdrawal and to establish consistent MOUD. Advised on methadone dosing and called and sent paperwork to local opioid treatment program for patient to continue treatment the next day and establish regular dosing.
Withdrawal management	Patient with OUD and alcohol use disorder admitted for acute alcohol and benzodiazepine withdrawal. ACS clinician advised to start patient on acamprosate and coordinated with patient’s primary care provider to help plan a trial of injectable buprenorphine on close outpatient follow-up.
MOUD dosing guidance	ACS clinician provided guidance to inpatient provider on management of methadone and benzodiazepine dose.

Following the creation of the CAC, the overall perception of addiction care at the hospital in the community has notably been more positive per anecdotal reporting shared during monthly coalition meetings and documented in coalition meeting minutes. Clinicians noted anecdotal evidence of positive outcomes, including multiple patients’ initiation of outpatient MOUD treatment upon discharge from the hospital as well as documented transitions to outpatient care. Hospital providers who used the CAC services also reported feeling more confident about their treatment of PWUD, specifically around the initiation of MOUD, and especially methadone. Although this intervention did not include an assessment of long-term impacts on patients or the hospital as a whole, previous research indicates that use of an Addiction Consult Service can improve life expectancy, reduce healthcare costs, and reduce both hospitalizations and overdose deaths [9]. No adverse or unintended consequences were observed during this time. Hospital leadership was invited to attend coalition meetings where narratives describing the positive impacts of the CAC were shared. These positive narratives in part shaped hospital leadership’s commitment to sustaining the CAC.

### Sustainability

Following the completion of the HCS and cessation of its funding for the CAC, hospital leadership continued the CAC program with hospital-based funding. Data from the CAC program was subsequently used to apply for grants to develop additional addiction medicine resources in community and rural hospitals. In January 2024, the hospital was awarded a grant through the Massachusetts Bureau of Substance Addiction Services. This funding will expand the CAC program to two additional community hospitals, support in-person and telehealth addiction consultation services (which would be billable services that might generate revenue for the service), and integrate hospital-based recovery coaching. To increase sustainability independent of grant funding, next steps for the program will include CAC telehealth patient consultations to generate revenue.

## Discussion and conclusions

The CAC service was developed through the application of the Communities That Heal intervention model. This framework is a blueprint for communities to make data-driven decisions in designing interventions responsive to community needs. Access to low-barrier MOUD and real-time training and technical assistance to hospital providers can reduce overdose rates, stigma, and complications from drug use while lowering rates of patient-directed discharges [5, 6]. 

Unlike traditional ACS models, the CAC has a key difference in that consults are provided for hospital staff by outside clinicians, thus recognizing that addiction medicine expertise can reside outside of the traditional hospital setting. They offer real-time, virtual consultations, and are a model for other rural communities without access to an inpatient addiction consult service or clinical expertise. Furthermore, offering telehealth consultations for hospital providers was an effective measure to overcome barriers related to transportation and clinician capacity. The CAC service is an innovative model mobilizing community addiction clinicians to expand addiction services in a rural hospital setting.

The CTH intervention is a community-responsive framework that allows communities to evaluate and implement targeted health interventions. The CTH framework has been replicated by HCS across four states to implement evidence-based strategies to reduce opioid-related overdose deaths. When applied in the FCNQ cluster community, the creation of a telehealth addiction consultation service to support clinical practice was an answer to the calls for more rural-specific studies and interventions to increase MOUD services [6]. The success of the CAC demonstrates the feasibility of community-engaged interventions that respond to the gaps in rural SUD treatment.

### Limitations

A limitation of our study is the anecdotal nature of how we collected feedback on experiences with the CAC from hospital staff and community members. People with positive experiences may have been more likely to attend coalition meetings and provide feedback, reflecting reporting bias and selection bias. Researchers’ positionality and power dynamics may also have resulted in social desirability bias in feedback. In addition to collecting quantitative data on patient outcomes, future research should involve formal, anonymous feedback collection from hospital staff and community members on the CAC and should incorporate patient voice as well.

## Data Availability

No datasets were generated or analysed during the current study.

## Abbreviations

ACS: Addiction Consult Service
CAC: Community Addiction Consult
CTH: Communities That HEAL
FCNQ: Franklin County/North Quabbin
HCS: HEALing Communities Study
HEAL: Helping to End Addiction Long Term
HIPAA: Health Insurance Portability and Accountability Act
MOUD: Medications for Opioid Use Disorder
OUD: Opioid Use Disorder
PWUD: People Who Use Drugs
SUD: Substance Use Disorder
